# Mischinfektionen bei kontaktlinsenassoziierter mykotischer Keratitis mit *Pseudomonas* oder Akanthamöben

**DOI:** 10.1007/s00347-020-01207-1

**Published:** 2020-08-21

**Authors:** C. J. Farah, B. Seitz, L. Hamon, C. Sourlis, L. Daas

**Affiliations:** Klinik für Augenheilkunde, Universitätsklinikum des Saarlandes UKS, Kirrberger Str. 100, Gebäude 22, 66421 Homburg/Saar, Deutschland

**Keywords:** Hornhaut, Kontaktlinse, Pilz, Keratitis, Konfokale Mikroskopie, Cornea, Contact lens, Fungal keratitis, Confocal microscopy

## Abstract

Kontaktlinsenassoziierte Keratitiden werden immer häufiger. Die mykotische Keratitis ist ein relativ seltenes, aber sehr ernst zu nehmendes Krankheitsbild. Meist wird im Frühstadium eine falsche Diagnose gestellt und dadurch die adäquate Therapie verzögert. Bei der therapierefraktären kontaktlinsenassoziierten mykotischen Keratitis können nicht selten auch Koinfektionen oder Superinfektionen bestehen. Wir stellen 2 Patienten mit initial unklarer Keratitis vor, bei denen eine Mischinfektion der mykotischen Keratitis mit *Pseudomonas aeruginosa* bzw. Akanthamöben nachgewiesen werden konnte. In beiden Fällen war die zeitnahe perforierende Excimerlaser-Keratoplastik mit Einzelknüpfnähten und adäquater Lokaltherapie über 8 Wochen therapeutisch erfolgreich.

## Abstract (en)

Contact lens-associated keratitis is becoming increasingly more frequent. Fungal keratitis is a relatively rare clinical picture but must be taken very seriously. Especially in the early stages of the disease, it may be clinically misdiagnosed and adequate treatment is therefore delayed. In treatment-resistant contact lens-associated fungal keratitis, coinfections or superinfections can occur. We present two patients with an initially unclear keratitis, in whom a fungal keratitis with coinfection of *Pseudomonas aeruginosa* and *Acanthamoeba*, respectively, could be confirmed. In both cases an urgent excimer laser penetrating keratoplasty with interrupted sutures and adequate local topical treatment for 8 weeks was successful.

Pilzkeratitiden sind ein relativ seltenes, aber sehr ernst zu nehmendes Krankheitsbild. Die korrekte Diagnose wird in Deutschland initial nur bei 1/5 der Fälle gestellt [1, 10]. Die Gefahr für das Auge ist groß, denn nicht adäquat behandelt, kann es zu Hornhautvernarbungen, Augenperforationen oder Endophthalmitiden kommen, was die visuelle Prognose erheblich reduziert und den Erhalt des Auges unsicher macht. Eine perforierende Keratoplastik ist im Verlauf nicht ungewöhnlich. In dem Deutschen Pilz-Keratitis-Register erhielten 65,7 % der eingeschlossenen Patienten im Verlauf der Krankheit eine perforierende Keratoplastik [10].

Die klinische Diagnosestellung einer mykotischen Keratitis stellt uns besonders bei atypischem Krankheitsbild vor Herausforderungen. Eine Kontaktlinsenanamnese lässt uns manchmal a priori zu Unrecht eine solitäre Akanthamöbenkeratitis vermuten. Aufgrund der möglichen Koinfektion sind wir der Meinung, dass alle diagnostischen Modalitäten – Polymerasekettenreaktion (PCR), In-vitro-Kultivierung, Histologie und in vivo konfokale Mikroskopie – parallel durchgeführt werden sollten [4, 11]. Bei der therapierefraktären kontaktlinsenassoziierten mykotischen Keratitis muss man nicht selten auch auf Koinfektionen oder Superinfektionen gefasst sein [8, 9]. Bei Koinfektionen ist es meistens problematisch, rechtzeitig die adäquate Therapie einzuleiten.

## Klinischer Befund

### Patientin 1.

**Figure 1:**
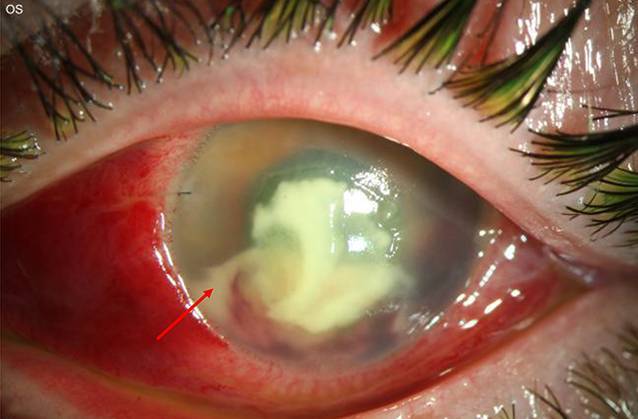

Eine 37-jährige Kontaktlinsenträgerin stellte sich zur Mitbeurteilung mit seit 5 Tagen zunehmender Visusminderung und Schmerzen bei therapierefraktärer Keratitis in unserer Hochschulambulanz vor. Die Patientin hatte keine Haustiere, und anamnestisch war Kontakt mit kontaminiertem Wasser nicht bekannt. Eine Kombinationstherapie von Tobramycin und Dexamethason-Augentropfen sowie lokale und systemische Aciclovir-Gabe (5-mal 400 mg pro Tag) war extern eingeleitet worden. Spaltlampenbiomikroskopisch zeigten sich am linken Auge ein großes stromales Infiltrat mit Epitheldefekt und die Vorderkammer mit einem für mykotische Keratitiden typischen pyramidenförmigen Hypopyon (Abb. 1, Bild nach Vorderkammerspülung).

### Patientin 2.

**Figure 2:**
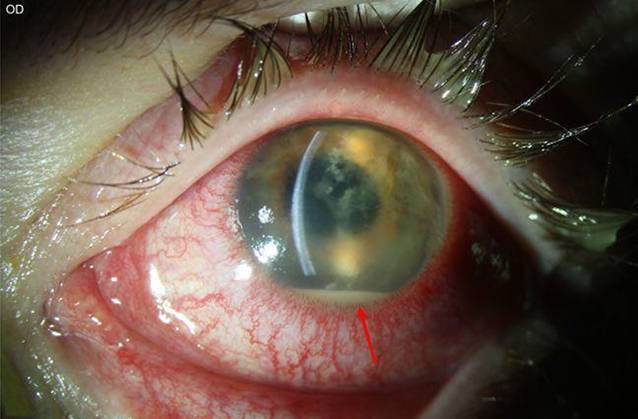

Eine 25-jährige Kontaktlinsenträgerin stellte sich zur Mitbeurteilung mit seit 4 Wochen bestehender Visusminderung, Lichtempfindlichkeit und Schmerzen bei therapierefraktärer Keratitis in unserer Hochschulambulanz vor. Die Patientin geht in ihrer Freizeit oft reiten, und anamnestisch war kein Kontakt mit kontaminiertem Wasser bekannt. Eine Kombinationstherapie von Moxifloxacin, Polymyxin B, Neomycin und Gramicidin, Dexamethason und antimykotischen (Voriconazol) Augentropfen war extern eingeleitet worden. Spaltlampenbiomikroskopisch zeigte sich am linken Auge eine diffuse interstitielle Keratitis mit ringförmigen multifokal akzentuierten Infiltraten, retrokornealen Beschlägen und einem 2‑mm-Hypopyon (Abb. 2).

## Diagnose

**Figure 3:**
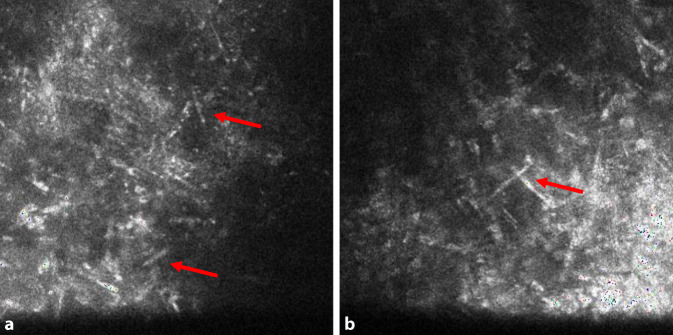

Bei *Patientin 1* betrug bei der Aufnahme der bestkorrigierte Visus (BCVA) Handbewegung am betroffenen linken Auge und 0,8 am gesunden rechten Auge. In der Sonographie bestand kein Anhalt für eine Endophthalmitis. Es zeigten sich in der konfokalen Mikroskopie hyperreflektive Strukturen (Abb. 3a, b) die für eine mykotische Keratitis sprechen. Eine Hornhautabrasion, Vorderkammerspülung mit Medikamenteingabe (Amphotericin B) und diagnostische Vorderkammeraspiration wurden am gleichen Tag durchgeführt. Bei therapierefraktärem Hypopyon und starkem Verdacht auf Pilzkeratitis führten wir am 3. Tag der Aufnahme eine Keratoplastik à chaud durch. Die PCR war 4 Tage nach der stationären Aufnahme negativ im Vorderkammeraspirat, aber positiv für Pilze im Hornhautabradat. Auch *Pseudomonas aeruginosa* konnte zeitnah in der In-vitro-Kultivierung nachgewiesen werden. Die PCR der Kontaktlinse und Kontaktlinsenbehälter sicherten am nächsten Tag eine Mischinfektion von *Pseudomonas aeruginosa* und *Candida parapsilosis*.

**Figure 4:**
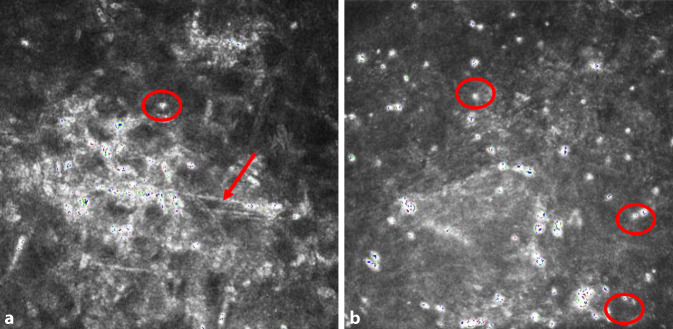

Bei *Patientin 2* betrug am Tag der stationären Aufnahme nach einer 4‑wöchigen Vorbehandlung der bestkorrigierte Visus (BCVA) 0,1 am betroffenen linken Auge und 1,0 am gesunden rechten Auge. Bis zum Tag der Aufnahme konnten extern keine Erreger nachgewiesen werden. In der Sonographie bestand kein Anhalt für eine Endophthalmitis. In der konfokalen Mikroskopie konnten sowohl hyperreflektive Linien wie bei mykotischer Keratitis als auch *Akanthamöben-Zysten* nachgewiesen werden (Abb. 4a, b). Eine Hornhautabrasion, Vorderkammerspülung mit Medikamenteingabe (Amphotericin B) und diagnostische Vorderkammeraspiration konnten am nächsten Tag durchgeführt werden. Die PCR sicherte eine Mischinfektion von Pilz (Pilz-DNA nicht weiter differenzierbar) und Akanthamöben im Hornhautabradat. Das Vorderkammeraspirat war negativ, und die In-vitro-Kultivierung konnte 4 Wochen nach einer durchgeführten Keratoplastik à chaud kein Wachstum nachweisen.

## Therapie und Verlauf

Beide Patientinnen erhielten nach intensiver lokaler und systemischer antibakterieller und antimykotischer Therapie eine Excimerlaser-Keratoplastik à chaud (Transplantatdurchmesser bei Patientin 1: 8,0/8,1 mm, bei Patientin 2: 8,5/8,6 mm; mit 24 Einzelknüpfnähten in beiden Augen) mit mehrmaliger Vorderkammerspülung, Medikamenteingabe (Amphotericin B) und ggf. antiamöboider Therapie im Verlauf.

### Patientin 1

**Figure 5:**
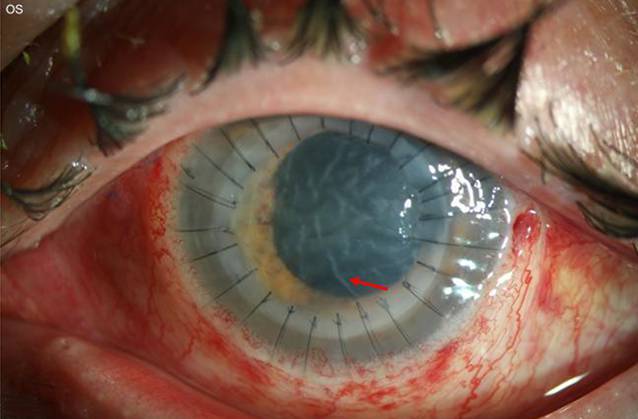

Die lokale Therapie bestand in unserer Klinik aus Voriconazol 2 %, Amphotericin B 1 % und Fortified-Augentropfen (Tobramycin und Cefazolin 5 %), da beide Keime (*Candida* und *Pseudomonas*) dagegen sensibel waren. Die systemische Therapie bestand aus Voriconazol p.o. 200 mg 1‑0‑1, Vancomycin i.v. 1 g 1‑0‑1, Ceftazidim i.v. 2 g 1‑1‑1. Die Patientin wurde bei persistierendem Epitheldefekt mit einer Amnionmembrantransplantation als Patch entlassen. Die lokalen antimykotischen und Fortified-Augentropfen wurden über mehr als 2 Monate fortgesetzt mit langsam ausschleichender lokaler und milder systemischer Kortikosteroidtherapie (Therapiestart mit 100 mg oral mit Abstufung 20 mg alle 2 Tage) zur Nachbehandlung der Keratoplastik (Abb. 5).

### Patientin 2

**Figure 6:**
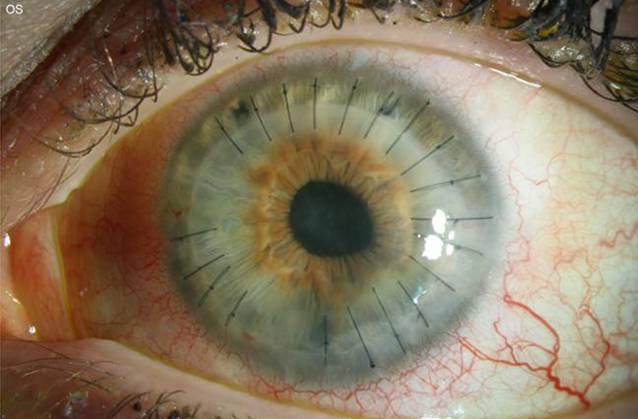

Die lokale Therapie bestand in unserer Klinik aus Voriconazol 2 %, Natamycin 5 %, Amphotericin B 1 %, Polyhexanid, Propamidine isethinate und einem Kombinationspräparat aus Neomycin‑, Polymyxin B- und Bacitracin/Gramicidin-Augentropfen. Die systemische Therapie bestand aus Voriconazol 200 mg 1‑0‑1 und milden Kortikosteroiden (100 mg mit Abstufung 20 mg alle 2 Tage) als Nachbehandlung der Keratoplastik (Abb. 6). Die lokale Triple-Therapie (Polyhexanid, Propamidine isethinate, Amphotericin B 1 %) wurde über 2 Monate fortgesetzt.

## Diskussion

Diese Doppelkasuistik betont die breite Differenzialdiagnose und die daraus potenziell resultierenden therapeutischen Schwierigkeiten bei kontaktlinsenassoziierten Keratitiden. Fehldiagnosen sind aufgrund atypischer Erscheinungsbilder in der frühen Phase häufig. Jedoch gibt es einige klinische Anzeichen, die den Augenarzt warnen sollten. Eine gründliche Anamnese des Kontakts mit Tieren oder kontaminiertem Wasser steigert die Angst vor Pilz- oder Akanthamöbeninfektionen. Eine Kontaktlinsenanamnese lässt uns manchmal a priori zu Unrecht eine solitäre Akanthamöbenkeratitis vermuten. Eine sehr schnell fortschreitende eitrige stromale Keratitis deutet auf eine *Pseudomonas*-Infektion hin [3]. Pilzkeratitiden können als haloförmiges Infiltrat mit möglichen Satellitenläsionen erscheinen [1, 4, 10]. Zu diesem Krankheitsbild sind das pyramidenförmige Hypopyon und das Hornhautinfiltrat mit intaktem Epithel assoziiert, in Deutschland auch als BB-1- und BB-2-Zeichen nach Behrens-Baumann bekannt [2, 11]. Aus unserer Erfahrung ist der Vorderkammerreizzustand ein kritisches prognostisches Kriterium bei allen mykotischen Keratitiden.

Die wiederkehrenden Akanthamöbeninfektionen werden als Frühzeichen durch das sog. „Dirty-Epithelium“ gekennzeichnet, Schmerzen sind typischerweise stark, aber uneinheitlich [5].

Eine angemessene Behandlung kann nur durch ein systematisches Vorgehen erreicht werden. Aufgrund der möglichen Mischinfektion sind wir der Meinung, dass alle diagnostischen Modalitäten – Polymerasekettenreaktion (PCR), In-vitro-Kultivierung, Histologie und in vivo konfokale Mikroskopie am Aufnahmetag – parallel durchgeführt werden sollten [4, 8, 11]. Konfokale Mikroskopie ist nicht als ein alleinstehendes diagnostisches Verfahren anzusehen, sondern als Ergänzung zu dem Spektrum der diagnostischen Modalitäten besonders bei atypischen Hornhautbefunden. Die Interpretation der konfokalen Mikroskopie hängt von der Expertise des Untersuchers ab [6].

Wertschätzendes interdisziplinäres Zusammenarbeiten mit der Mikrobiologie ist unerlässlich zur Identifizierung der Pathogene und Anpassung der Therapie. Bei Verdacht auf Pilzinfektion (mit unbekanntem Erreger) sollte topisches Voriconazol 2 % als Primärbehandlung eingeleitet werden, denn der antimykotische Wirkstoff kann auch gegen andere Erreger wie Akanthamöben wirken [1, 7]. In beiden Fällen war die zeitnahe perforierende Excimerlaser-Keratoplastik mit Einzelknüpfnähten und adäquater Lokaltherapie über 8 Wochen therapeutisch erfolgreich.

## Fazit für die Praxis

Bei jeder unklaren oder therapieresistenten kontaktlinsenassoziierten Keratitis sollte an eine Mischinfektion gedacht werden, und eine Polymerasekettenreaktion (PCR), In-vitro-Kultivierung, Histologie und in vivo konfokale Mikroskopie sollten am Aufnahmetag parallel durchgeführt werden.
